# Valence Encoding Signals in the Human Amygdala and the Willingness to Eat

**DOI:** 10.1523/JNEUROSCI.2382-19.2020

**Published:** 2020-07-01

**Authors:** Lena J. Tiedemann, Arjen Alink, Judith Beck, Christian Büchel, Stefanie Brassen

**Affiliations:** Department of Systems Neuroscience, University Medical Centre Hamburg-Eppendorf, Hamburg, D-20246, Germany

**Keywords:** fMRI, food valence, multivariate pattern similarity, willingness to eat

## Abstract

One of the strongest drivers of food consumption is pleasure, and with a large variety of palatable food continuously available, there is rarely any necessity to eat something not tasty. The amygdala is involved in hedonic valuation, but its role in valence assignment during food choices is less understood. Given recent evidence for spatially segregated amygdala signatures encoding palatability, we applied a multivariate approach on fMRI data to extract valence-specific signal patterns during an explicit evaluation of food liking. These valence localizers were then used to identify hedonic valuation processes while the same healthy human participants (14 female, 16 male; in overnight fasted state on both scanning days) performed a willingness-to-eat task in a separate fMRI measurement. Valence-specific patterns of amygdala signaling predicted decisions on food consumption significantly. Findings could be validated using the same valence localizers to predict consumption decisions participants made on a separate set of food stimuli that had not been used for localizer identification. Control analyses revealed these findings to be restricted to a multivariate compared with a univariate approach, and to be specific for valence processing in the amygdala. Spatially distributed valuation signals of the amygdala thus appear to modulate appetitive consumption decisions, and may be useful to identify current hedonic valuation processes triggering food choices even when not explicitly instructed.

**SIGNIFICANCE STATEMENT** The expectation of tastiness is a particularly strong driver in everyday decisions on food consumption. The amygdala is important for hedonic valuation processes and involved in valence-related behavior, but the relationship between both processes is less understood. Here, we show that hedonic values of food are represented in spatially distributed activation patterns in the amygdala. The engagement of these patterns during food choices modulates consumption decisions. Findings are stable in a separate stimulus set. These results suggest that valence-specific amygdala signals are integrated into the formation of food choices.

## Introduction

Consumption decisions are driven by several different factors, including body energy levels (Anderberg et al., 2016), social environment (Higgs and Thomas, 2016), or the nutritive properties of food (Suzuki et al., 2017). Yet one of the strongest drivers of food consumption is pleasure (Berthoud, 2011), and understanding mechanisms and modulators of hedonic eating has become a major research focus in the face of a steady increase of overweight and obesity (Berthoud et al., 2017). Food intake is regulated via metabolic signals which are integrated in homeostatic brain structures, such as the hypothalamus, as well as nonhomeostatic regions of the dopaminergic and opioidergic systems (Berthoud, 2011; Tellez et al., 2013; Stuber and Wise, 2016). Previous research has unraveled the neurocircuits underlying food decisions (Hare et al., 2011; Rangel, 2013), and those underlying valence coding (Stice et al., 2013; Jin et al., 2015), but little is known about how implicit valuation processes modulate food choices as probably occurring during everyday consumption decisions.

The amygdala with its reciprocal connections to the sensory, prefrontal, and reward systems (Sah et al., 2003; Janak and Tye, 2015) is a key region in valence assignment (Sergerie et al., 2008) and in engaging valence-specific behavioral responses (Pessoa, 2010; O'Neill et al., 2018; Pignatelli and Beyeler, 2019). Given its central location within a dense neural network transferring food and feeding-relevant information (E. M. Kim et al., 2004; Peciña et al., 2006; Wassum et al., 2009; Berthoud, 2011; Stone et al., 2011; Sun et al., 2014), it appears to be the optimal target when studying the mechanisms of valence assignment on food choices. For instance, in rodents, variable palatability is encoded through specific amygdala signals, with similarly liked foods evoking comparable response patterns (Fontanini et al., 2009; Sadacca et al., 2012). Imaging studies in humans and rodents showed that the amygdala is involved in assessing the valence and salience of learned food cues (Andermann and Lowell, 2017) and in both the anticipation as well as the consumption of food (O'Doherty et al., 2001, 2002) and odors (Sorokowska et al., 2016). Furthermore, intracranial recoding from amygdala neurons indicates food values to be encoded automatically, regardless of the task (Mormann et al., 2017). Together, these findings underline the amygdala as a key candidate in the investigation of hedonically driven consumption decisions.

Based on recent animal literature (Kim et al., 2016; Beyeler et al., 2018; O'Neill et al., 2018), it can be questioned whether the complexity of valence can be represented by averaged amygdala signals or may better be represented in a distributed fashion. Accordingly, pattern-based multivariate analysis (Kriegeskorte et al., 2008) has been used to investigate amygdala activity during odor perception. Findings implicate that spatial amygdala patterns encode the entire dimension of valence, ranging from pleasantness to unpleasantness (Jin et al., 2015). This fits with studies in rodents showing that amygdala subregions contain distinct populations of neurons activated by negative or positive stimuli (Kim et al., 2016; Beyeler et al., 2018; O'Neill et al., 2018), as recently also shown in the context of palatability (Wang et al., 2018).

In the present study, we followed a two-step approach: First, we used multivariate representational similarity analysis (RSA) to identify valence-specific amygdala patterns while participants explicitly evaluated the general likeability of visual food stimuli. Second, we used extracted multivoxel valence localizers to map valence processing during consumption decisions in a separate measurement to predict food choices of the same but also of a separate set of food stimuli. Our findings show that multivoxel amygdala patterns represent food valence during explicit valuation and that these valence signals modulate consumption decisions. Analyses of consumption decisions on food stimuli from an independent stimulus set underline the validity of spatial valence patterns regardless of stimulus identity. Control analyses demonstrate that these findings were restricted to a multivariate compared with a univariate approach, and were specific for valence processing (as opposed to general choice value) and the amygdala (as opposed to the vmPFC). These data suggest that consumption decisions are accompanied by valence processing in the amygdala that modulates food decisions.

## Materials and Methods

### 

#### 

##### Participants

Thirty volunteers (mean ± SD age: 25.07 ± 2.5 years; range = 21–30 years; 14 female) participated in the present study. Participants were recruited via online announcements and existing databases. Inclusion criteria comprise normal weight (WHO guidelines: waist circumference ≤ 94 cm for men and ≤ 80 cm for women), the absence of current or previous psychiatric or neurologic disorders, acute or chronic physical illness, current psychopharmacological medication, adherence to a specific diet or severe food allergies, as well as MR-specific exclusion criteria. No participant had deliberately tried to change her/his eating behavior or body status in the 6 months preceding the experiment.

Research was conducted in accordance with the Helsinki Declaration and was approved by the local ethics committee. All subjects were financially compensated for participation and gave written informed consent before the experiment.

##### Experimental design

All participants attended two separate study days (at least 1 week apart). On each scanning day, participants arrived in the morning between 7:30 A.M. and 10:45 A.M. after an overnight fast of at least 10 hours. Anthropometric measurements were taken, and participants rated their current feelings of hunger on a scale from 0 (“not hungry at all”) to 10 (“extremely hungry”). To confirm fasted state in all participants, blood samples were taken on both study days to analyze blood glucose levels.

**Figure 1.**
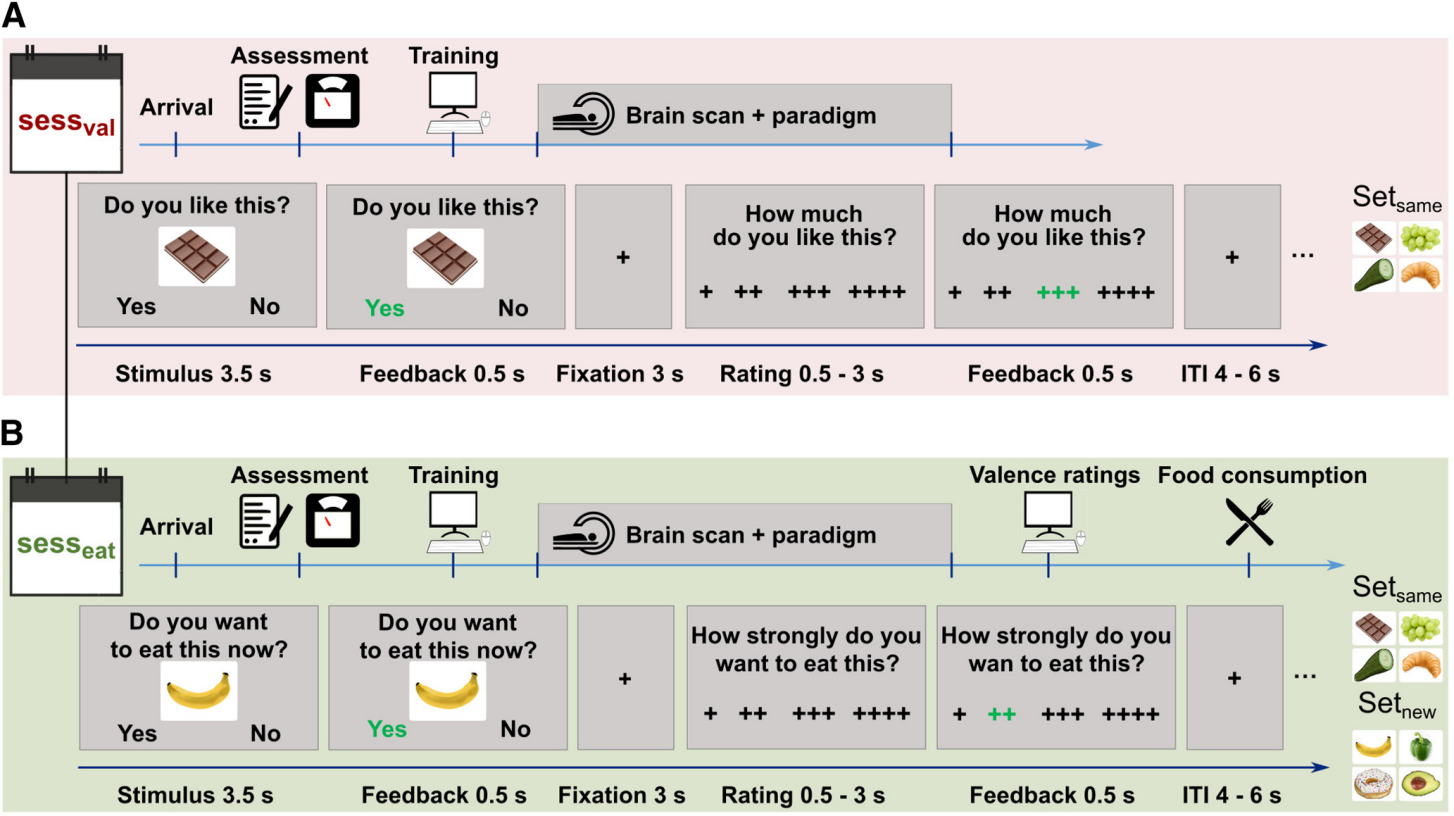
Study design and paradigms. ***A***, During the first scanning day (sess_val_), participants rated the hedonic valence of food items from one of two stimulus sets without the option to consume the food. ***B***, During the second day (sess_eat_), participants had to indicate their WTE food that had been presented on the first scanning day (set_same_) intermixed with food items from the separate stimulus set that was not shown on sess_val_ (set_new_). This session was followed by valence ratings and a consumption phase. ITI, Intertrial interval.

On the first study day, each participant was presented with one of two sets of visual food stimuli and was asked to evaluate the general likeability of these food items (Fig. 1*A*, sess_val_). Each set depicted 70 distinguishable food pictures (e.g., Set 1: apple; Set 2: pear). The allocation of sets was randomly counterbalanced across participants. Predefined selection criteria (e.g., with respect to the amount of fruits, vegetables, sweets, etc.) ensured that sets are comparable in critical aspects. This was confirmed by a validation study in an independent sample of 16 participants showing that the two sets did not differ significantly (all *p* > 0.18) regarding macronutrient composition, caloric content, valence, and picture salience.

On the second study day, all participants were then presented with the items of both sets with stimuli randomly intermixed. Participants were now asked to indicate their willingness to eat (WTE) each of these 140 food items at that very moment (Fig. 1*B*, sess_eat_). That is, during the WTE task, each participant decided about food items shown on the first study day (set_same_) as well as new food items (set_new_). Ecological validity of single-trial decisions was ensured by informing participants beforehand that after the scanning session they have to eat a mouthful of a randomly selected food item they indicated they want to eat. This food consumption was preceded by another likability valuation of all 140 food items to obtain valence ratings of all food items. Likeability and decision tasks are adapted versions of previously established behavioral paradigms (Finlayson et al., 2007; Hare et al., 2011; Tibboel et al., 2011).

Timing and task structure were identical for both fMRI paradigms (Fig. 1). In each trial, stimuli were presented for 4 s, during which participants first gave binary answers regarding the valence (sess_val_; Fig. 1*A*) or WTE (sess_eat_; Fig. 1*B*) of the food. Second, they further detailed their answers which allowed for the construction of an 8-level scale, ranging from 1, the lowest valence/willingness (“no,” “– – – –”), to 8, the highest valence/WTE (“yes,” “+ + + +”).

Visual food stimuli (e.g., chocolate, ice cream, tomatoes, apples, carrots) had been selected from the Internet and contained only the food, without brand names or packaging. They covered a wide range of macronutrient composition and caloric content (sugar: 0–78 g/100 g, complex carbohydrates: 0–66.2 g/100 g, protein: 0.2–25.4 g/100 g, fat: 0–62 g/100 g, absolute calories: 16–666 kcal/100 g). All pictures had a size of 400 × 400 pixels and were centrally presented on a white background.

##### Statistical analyses

Behavioral and metabolic data were analyzed using paired and one-sample *t* tests as well as Pearson correlations. One-sample *t* tests were used for group statistics on neural data. To account for multiple comparisons, results were corrected using Bonferroni and familywise error correction (FWE) with *p* < 0.05. Specifically, correlations between macronutrients and valence/WTE ratings were corrected for five comparisons each (corrected *p* value: 0.01), RSAs were corrected for two ROIs (corrected *p* value: 0.025), and predictions of WTE were corrected for two ROIs and two stimulus sets (corrected *p* value: 0.0125).

##### (f)MRI data acquisition and preprocessing

All imaging data were acquired on a Siemens Trio 3T scanner using a 32-channel head coil. Functional data were obtained using a multiband EPI sequence. Each volume contained 60 slices (voxel size 1.5 × 1.5 × 1.5 mm) at an oblique orientation of 30° to the anterior commissure–posterior commissure axis (TR = 2.26 s, TE = 30 ms, flip angle = 80°, FOV = 225 mm, multiband mode CMRR, number of bands: 2). Additional MPRAGE structural images were acquired on both scanning days for functional preprocessing (240 slices, voxel size 1 × 1 × 1 mm).

Structural and functional data were analyzed using SPM12 (Welcome Department of Cognitive Neurology, London) and custom scripts in MATLAB (version 2017a, The MathWorks). All functional volumes were corrected for rigid body motion and susceptibility artifacts (“realign and unwarp”). After coregistration with individual structural images, functional images were spatially normalized. For conventional univariate analysis, data were smoothed with a 4 mm FWHM isotropic Gaussian kernel.

##### ROI definition

ROIs for the bilateral amygdala and the occipital pole were defined in MNI space based on the FSL Harvard Oxford structures atlas (thresholded at 50% probability, Harvard Center for Morphometric Analysis; https://fsl.fmrib.ox.ac.uk/fsl/fslwiki/Atlases). For the vmPFC, a sphere with 20 mm radius was used that was centered on the peak voxel (−2, 46, −8) derived from 199 imaging studies reporting “vmPFC,” as determined by a meta-analysis conducted on the www.neurosynth.org platform (status 15/04/2020).

##### Univariate fMRI analyses

Several GLMs were constructed for the univariate analysis of imaging data. The first GLM tested for regions that show valence-related activation at sess_val_ by modeling the onsets of the 70 food items as a single regressor together with a parametric modulator coding for the 8 point valence ratings. Two further GLMs tested for activation that encodes individuals' WTE values on sess_eat_ for stimuli of the set_same_ and set_new_ by modeling the 8 point WTE ratings as parametric modulators of food-related BOLD responses. Contrast images were entered into second-level one-sample *t* tests. Imaging findings were reported when passing small-volume corrections at an FWE-corrected threshold of *p* < 0.05 or when passing a whole-brain corrected cluster-threshold of FWE < 0.05 (cluster forming threshold *p* < 0.001 uncorrected).

##### Multivariate fMRI analyses

First, RSA was applied on fMRI data from sess_val_ to test and extract valence-specific pattern localizers in the amygdala and, as a control region, in V1. For this, a first-level GLM was calculated in which onsets of food stimuli with the same valence value were modeled as separate regressors convolving δ functions with a canonical hemodynamic response function on the unsmoothed functional data. Beta estimates were then extracted from each voxel within the bilateral ROIs, resulting in one vector summarizing all voxels per valence value. Multivoxel data vectors corresponding to each valence value were then used to create an 8 × 8 subjectwise representational dissimilarity matrix (RDM; see Fig. 3*A*) per brain region through correlating each pair of valence patterns (measured as 1 – Pearson's *r*). The correspondence between neural and behavioral coding of valence was then examined using Spearman's rank correlation coefficients computed between these neural RDMs for brain patterns and distances in behavioral valence ratings (model RDM; see Fig. 3*A*). Finally, one-sample *t* tests on resulting Fisher *z*-transformed correlation coefficients were used to test the significance of brain-behavior correspondence across subjects.

In the next step, we tested whether amygdala signaling from sess_eat_, which corresponds to valence-specific activation patterns identified in sess_val_, predicts the WTE by using a Least Squares All procedure (Mumford et al., 2012) on items from set_same_ and set_new_ separately. To this end, a first-level GLM was conducted modeling each stimulus onset from sess_eat_ as a separate regressor on the unsmoothed functional images. Beta values were extracted from each voxel within the amygdala ROI, resulting in one vector summarizing all voxels per food item. Multivoxel data vectors of each item were then used to create a 70 × 8 subjectwise valence specificity matrix (see Fig. 4*A*) through correlating this amygdala ensemble activity per food item with each vectorized valence pattern from sess_val_. To statistically test the correspondence between neural valence specificity and WTE, Spearman's rank correlation coefficients were computed between these neural pattern similarity indices and a WTE matrix representing distances in behavioral WTE ratings. This tested for the assumption that similar valence-specific amygdala patterns correspond to similar WTE ratings and vice versa. One-sample *t* tests on resulting Fisher *z*-transformed correlation coefficients were used to test the significance of brain-behavior correspondence across subjects.

In a set of control analyses, we aimed to further elucidate the specific role of the amygdala for encoding the valence of food items at time of choice. For this purpose, we repeated the complete multivariate pattern analysis described above in the vmPFC as a well-known region involved in general choice behavior (Levy and Glimcher, 2012; Bartra et al., 2013). In addition, we reran RSA on amygdala data from sess_eat_, but using WTE instead of valence ratings for vectorizing brain data and modeling of behavioral data.

## Results

### Behavioral data

**Figure 2.**
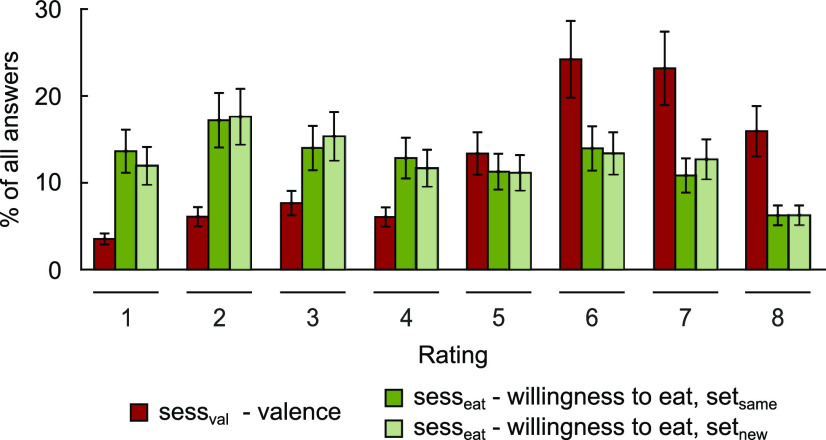
Distribution of ratings. Group means and SEM of valence and WTE ratings, ranging from 1 (“not at all”) to 8 (“very much”).

sess_val_ was followed by sess_eat_ after an average duration of 14.13 d (SD = 6.55 d). Fasting glucose levels confirmed fasting state in all participants on both study days (glucose sess_val_: 4.74 ± 0.46 mmol l^−1^; sess_eat_: 4.76 ± 0.41 mmol l^−1^). Glucose levels, fasting duration (fasting duration sess_val_: 12.85 ± 1.48 h; sess_eat_: 12.85 ± 1.29 h), and hunger ratings (hunger sess_val_: 4.00 ± 2.45; hunger sess_eat_: 4.17 ± 2.18) did not differ between the two study days (all *t*_(29)_ < 0.34, all Cohen's *d* < 0.063, all *p* > 0.733). Across participants, food items received a mean valence rating of 5.66 (SD = 0.55, Fig. 2). Participants disliked between 10.00% and 50.75% of the food items (mean = 23.31%), that is, between 7 and 35 food items. Valence ratings were not significantly correlated with caloric (*t*_(29)_ = 0.18, Cohen's *d* = 0.033, *p* = 0.858), sugar (*t*_(29)_ = 0.85, Cohen's *d* = 0.153, *p* = 0.409), complex carbohydrates (*t*_(29)_ = 0.32, Cohen's *d* = 0.057, *p* = 0.757), fat (*t*_(29)_ = 0.09, Cohen's *d* = 0.016, *p* = 0.932), or protein content (*t*_(29)_ = 2.62, Cohen's *d* = 0.478, *p* = 0.014, not significant after Bonferroni correction). Food items in set_same_ and in set_new_ did not differ with respect to valence and WTE ratings during sess_eat_ (mean ± SD valence: set_same_: 5.22 ± 0.55, set_new_: 5.28 ± 0.69, *t*_(29)_ = 0.85, Cohen's *d* = 0.155, *p* = 0.40; mean ± SD WTE: set_same_: 4.07 ± 0.80, set_new_: 4.14 ± 0.77, *t*_(29)_ = 0.85, Cohen's *d* = 0.154, *p* = 0.41; Fig. 2). In neither of the two sets was WTE correlated with any macronutrient or caloric content (all *t*_(29)_ < 1.62, all Cohen's *d* < 0.295, all *p* > 0.117), but it was significantly correlated with valence ratings across participants (mean ± SD valence × WTE in set_same_: 0.72 ± 0.10, *t*_(29)_ = 40.34, Cohen's *d* = 7.366, *p* < 0.001; valence × WTE in set_new_: 0.74 ± 0.07, *t*_(29)_ = 55.56, Cohen's *d* = 10.144, *p* < 0.001).

While hunger was not correlated with valence ratings during sess_val_ (*r* = 0.26, *p* = 0.17), there was a significantly positive correlation of hunger with WTE during sess_eat_ (*r* = 0.62, *p* < 0.001). Accordingly, correlations of hunger ratings with valence and WTE ratings differed significantly (*z* = 1.69, *p* = 0.046), indicating that hunger drove eating decisions but not general palatability valuation.

### Neural valence coding during likeability evaluation

**Figure 3.**
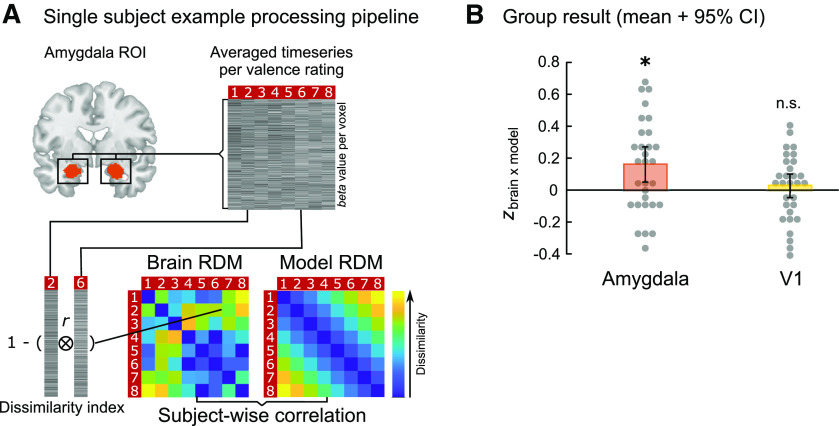
Valence coding in the amygdala. ***A***, Single-subject example processing pipeline of the RSA on data from sess_val_. ***B***, Group result: Mean and 95% CI of Fisher *z*-transformed correlation coefficients between brain and model RDMs for the amygdala and, as a control, the primary visual cortex, overlaid on single-subject results. *p < 0.025 (Bonferroni-corrected for multiple comparisons). n.s., *p* = 0.676.

To evaluate valence specificity of activation patterns in the amygdala, brain RDMs describing the similarity of neural activations for each pair of valence ratings were compared with model RDMs that quantified the similarity of valence ratings in a linear fashion (Fig. 3*A*). A one-sample *t* test of Fisher *z*-transformed correlation coefficients revealed significant evidence for the differential coding of valence through spatially distributed patterns in the amygdala (*t*_(29)_ = 2.73, Cohen's *d* = 0.498, *p* = 0.011; *p* < 0.025 Bonferroni-corrected for multiple comparisons; Fig. 3*B*). Repeating this analysis in V1 as a control region for general visual stimulus effects (e.g., saliency) revealed no evidence for valence coding in this area (*t*_(29)_ = 0.42, Cohen's *d* = 0.077, *p* = 0.676; Fig. 3*B*).

Using a univariate approach where subjective valence values were included as parametric modulators of food onset regressors revealed positive valence coding in the precuneus, the ACC, and the vmPFC (Table 1; see Fig. 6). No significant activation was observed in the amygdala. Thus, whole spectrum valence information could be detected through multivariate patterns, but not as a strictly linear component of the averaged signal in the amygdala.

**Table 1.** Regions encoding valence ratings during sess_val_^a^

Region	Side	Clustersize *k*_E_	Peak MNIcoordinates			Peak *z*scores
			*x*	*y*	*z*	
Parametric modulation by valence rating						
vmPFC	L	665	−3	48	−6	4.87
ACC	R	101	2	35	−10	4.59
Precuneus	R	125	6	−52	14	4.70
	L	336	−8	−54	11	5.22

*^a^p* < 0.05, whole-brain FWE correction for multiple comparisons at the cluster level (cluster-forming threshold, *p* < 0.001 uncorrected).

**Table 2.** Regions encoding willingness ratings during sess_eat_^a^

Region	Side	Clustersize *k*_E_	Peak MNIcoordinates			Peak *z*scores
			*x*	*y*	*z*	
Parametric modulation bywillingness rating: set_same_						
Precuneus		179	0	−54	20	4.83
Middle frontal gyrus	R	165	40	48	8	4.29
dlPFC	L	1308	−40	40	12	5.44
vmPFC	L	3196	−8	36	−10	4.96
Middle cingulate gyrus	R	199	2	−10	35	5.02
Parametric modulation bywillingness rating: set_same_						
dlPFC	L	540	−44	41	18	5.55
vmPFC	R	2098	2	30	11	5.48
Precuneus		218	0	−54	20	4.55

*^a^p* < 0.05, whole-brain FWE correction for multiple comparisons at the cluster level (cluster-forming threshold, *p* < 0.001 uncorrected).

### Amygdala valence patterns during consumption decisions

**Figure 4.**
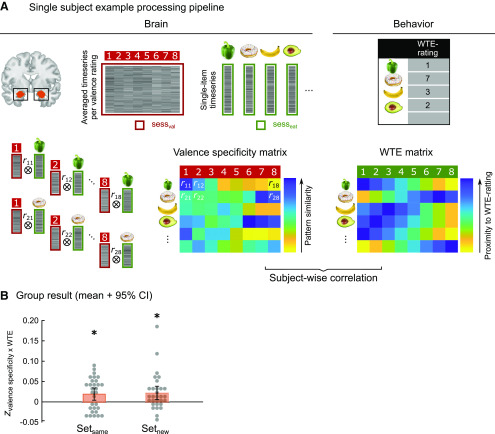
Prediction of WTE. ***A***, For each participant, single-trial amygdala valence patterns from sess_eat_ were correlated with each of the eight valence patterns identified in sess_val_ to construct an individual “Valence specificity matrix.” This brain matrix was correlated with a “WTE matrix” that described the WTE each food item using an 8 point gradient (reflecting monotonically decreasing similarity). ***B***, Mean and 95% CI of Fisher *z*-transformed correlation coefficients between valence specificity and WTE matrices, in the same set of items that were used to identify valence patterns on sess_val_, and in a new set of items, overlaid on single-subject results. *p < 0.0125 (Bonferroni-corrected for multiple comparisons).

Next, we tested whether amygdala signals in predefined valence patterns can predict participants' WTE on a separate study day. To this end, first we correlated single-item activation patterns from sess_eat_ with valence coding localizers from sess_val_ to decode valence-specific processing during consumption decisions. We hereby assumed that stronger correlations of itemwise activation patterns with a specific valence pattern from explicit valuation on sess_val_ indicate a similar valence processing on sess_eat_. We further assumed such implicit valence coding to predict WTE (i.e., higher neurally encoded valence values to promote stronger consumption decisions). To test this assumption, we specified an individual WTE matrix by arranging each food item's WTE rating within an 8 point distance gradient (Fig. 4*A*). Across participants, neural valence specificity and WTE matrices correlated significantly for set_same_ (*t*_(29)_ = 2.79, Cohen's *d* = 0.509, *p* = 0.009; *p* < 0.0125 Bonferroni-corrected; Fig. 4*B*), indicating a significant correspondence between neurally decoded valence values in the amygdala and decisions on food consumption. Importantly, this finding could be replicated in the separate stimulus set_new_ (*t*_(29)_ = 2.77, Cohen's *d* = 0.507, *p* = 0.010; *p* < 0.0125 Bonferroni-corrected; Fig. 4*B*); that is, valence localizers derived from the set_same_ could be used to decode valence processing and to predict the WTE of a new stimulus set of 70 food items. The strength of prediction in both sets was not related to individual hunger ratings (all *p* > 0.259).

### Control analyses

**Figure 5.**
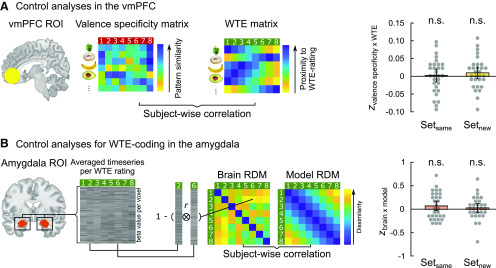
Control analyses. ***A***, Multivariate control analyses in the vmPFC did not reveal a significant impact of valence coding in this region on explaining variability in consumption decisions. ***B***, RSA analyses on amygdala data from sess_eat_ modeling WTE ratings did not provide evidence for spatial willingness pattern coding in the amygdala in either stimulus set. Error bars indicate means with 95% CI, overlaid on single-subject results. n.s., *p* = 0.234, *p* = 0.183, *p* = 0.168; *p* = 0.544.

In a set of control analyses, we investigated the specific importance of the amygdala for valence coding during food choices. First, we repeated the multivariate approach on data from sess_val_ and sess_eat_ in the vmPFC. As illustrated in Figure 5*A*, the critical analysis revealed that valence patterns in the vmPFC were not directly related to WTE behavior in either of the two stimulus sets (all *t*_(29)_ < 1.22, Cohen's *d* = 0.222, *p* = 0.234; Fig. 5*A*). Second, we explored whether WTE ratings themselves were spatially coded in the amygdala. For this purpose, we reran RSA (as illustrated in Fig. 3) on amygdala data from sess_eat_ using 8 point WTE ratings for vectorizing brain data and modeling of behavioral data (brain/model RDMs; Fig. 5*B*). Results did not provide evidence for spatial pattern coding of WTE in the amygdala for either stimulus set (set_same_: *t*_(29)_ = 1.41, Cohen's *d* = 0.258, *p* = 0.168; set_new_: *t*_(29)_ = 0.60, Cohen's *d* = 0.112, *p* = 0.544; Fig. 5*B*).

**Figure 6.**
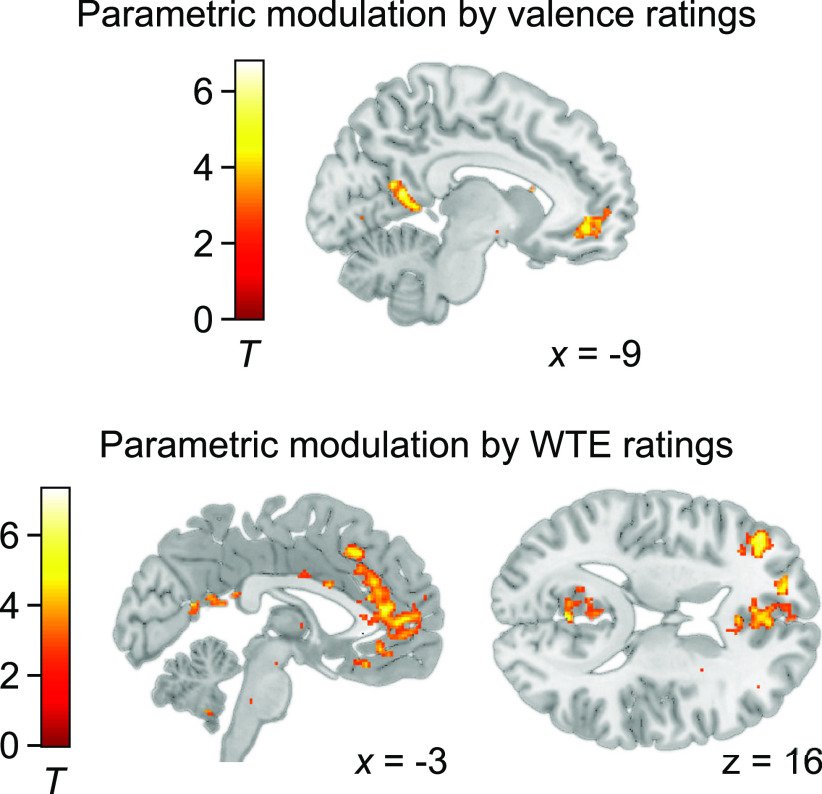
Univariate results: parametric modulation. Activation in the vmPFC (−3, 48, −6, peak *z* score = 4.87) and in the precuneus (right: 6, −52, 14, peak *z* score = 4.70; left: −8, −54, 11, peak *z* score = 5.22) correlated significantly with valence ratings across all participants (Table 1). Regions in which the correlation with the WTE values was significant across all participants included the vmPFC (−8, 36, −10, peak *z* score = 4.96), the dlPFC (−40, 40, 12, peak *z* score = 5.44), and the precuneus (0, −54, 20, peak *z* score = 4.83) (Table 2). Activations are overlaid on a standard anatomic template image (display threshold *p* < 0.001, uncorrected).

We finally returned to univariate analyses to examine parametric modulation of the BOLD response by WTE values. In accordance with large evidence on the brains' valuation network (Levy and Glimcher, 2012), whole-brain results demonstrated significant positive correlation in the vmPFC, the middle frontal gyrus, and the precuneus (Table 2; Fig. 6). No association between WTE and BOLD activity in the amygdala could be found in either of the two stimulus sets.

## Discussion

We identified distributed valence representations in the human amygdala and used these activation patterns to track effective valence processing during food choices. During the explicit pleasantness evaluation of food stimuli, hedonic values were differentially encoded across the whole spectrum through spatial patterns supporting an unidimensional bipolar model of valence encoding in the amygdala (Fontanini et al., 2009; Sadacca et al., 2012; Jin et al., 2015). Importantly, these valence-specific patterns could be used as localizers to decode valence values driving the WTE during consumption decisions. Findings could be validated in a separate stimulus set and were specific to multivariate fMRI signal patterns, the amygdala, and valence processing. These findings underline the impact of valence processing in the amygdala on consumption decisions in an implicit hedonic valuation context.

Our results demonstrate that unique, spatially segregated patterns in the amygdala encode the entire subjective valence dimension from pleasantness to unpleasantness. Specifically, the amygdala showed stronger pattern correlations among food cues of similar valence and weaker correlations among food cues of dissimilar valence. This regionally distributed valence coding is in line with previous findings in the human amygdala demonstrating multivoxel coding of differently valenced odors (Jin et al., 2015). Our data indicate that this dimensional coding is also relevant for valence processing in response to visual food cues, which agrees with recent findings from intracranial recordings in the human amygdala during visual food cue evaluation (Mormann et al., 2017). Our results generally are in line with large evidence on the amygdala's key role in valence processing as shown for various stimuli (Sergerie et al., 2008; Pessoa, 2010; Zerubavel et al., 2015). Amygdala valence computation (Seymour and Dolan, 2008) of food stimuli is thereby probably based on the integration of neural inputs signaling food memory (LaBar, 2007; Liénard et al., 2014) as well as sensory (Sah et al., 2003; Small, 2006), hedonic (Kim et al., 2004; Peciña et al., 2006; Castro and Berridge, 2017), and nutritive (Suzuki et al., 2017) stimulus properties.

Univariate analyses of fMRI signals in the amygdala did not reveal differential modulation by hedonic values in the present study. This fits with recent evidence in animals of topographically segregated neuron populations coding for positive and negative valence (Beyeler et al., 2016, 2018; Kim et al., 2016; O'Neill et al., 2018; Wang et al., 2018). In monkeys, for example, positive value-coding amygdala neurons have been shown to increase their firing rate to conditioned stimuli predicting reward but decrease their firing in response to aversive cues, whereas negative value-coding neurons demonstrate the opposite pattern (Belova et al., 2008). In rodents, rewarding stimuli primarily activated the posterior BLA, whereas aversive stimuli primarily activated the anterior BLA (Kim et al., 2016). Our findings extend evidence for a spatially distributed valence coding to the human amygdala.

Using multivoxel valence localizers from explicit likeability valuation, we were able to predict the current WTE different foods in a separate measurement. The strength of similarity between valence localizers and single-trial amygdala signal patterns during consumption decisions was thereby used to decode neural valence processing. Intriguingly, valence localizers helped to map such value processing during consumption decisions, even of food stimuli that were not involved in the identification of valence localizers. This not only validates our findings but also emphasizes a relative stability of valence coding in the amygdala regardless of stimulus identity. Recent findings demonstrate visual food stimuli to evoke amygdala responses, even in the absence of conscious awareness of food (Sato et al., 2019). Moreover, automatic value encoding in the amygdala in response to visual food stimuli has been identified previously also in valence-independent tasks (Mormann et al., 2017). Thus, one could speculate that amygdala responses on the sight of a visual food stimulus represent value information regardless of the task.

There is large evidence for the involvement of the amygdala in the modulation of valence-specific behavioral responses (O'Neill et al., 2018). Especially the opioid receptor-rich basolateral part is thought to convey the hedonic value of rewards into goal-directed actions probably based on incentive learning (Wang et al., 2005; Malvaez et al., 2019) and mediated via orbitofrontal pathways (Malvaez et al., 2019) as well as other brain systems typically involved in memory (Liu et al., 2018) and reward (Stuber et al., 2011). Valence assignment to food stimuli in the amygdala might thereby be passed to and processed in the orbitofrontal cortex and adjacent vmPFC, where the integration of valence together with other stimulus features might serve value computation and decision-making (Plassmann et al., 2010; Rangel and Hare, 2010; Kahnt et al., 2011; Dagher, 2012).

Importantly, WTE was not directly represented in amygdala activation signals, neither using univariate nor multivariate analyses. This indicates that neural valence processing explains a significant amount of variability in consumption decisions, but consumption decisions themselves are not specifically encoded in amygdala activation. In a similar vein, while our univariate analysis clearly demonstrates a strong involvement of the vmPFC in food choices, which is in line with large literature on decision-making (Levy and Glimcher, 2012), food valence signals in vmPFC patterns were not specifically related to food choices in the current study. These findings are probably due to the many other factors driving WTE decisions and modulating underlying neural valuation processes. For instance, recent data suggest a reinforcing value of food macronutrients, such as fat and carbohydrates that is processed via the striatum and the gut-brain axis and is independent of food liking (DiFeliceantonio et al., 2018; de Araujo et al., 2020). Moreover, food deprivation, as in our sample, has been discussed to promote a preference for high-calorie food items (Figlewicz, 2015; Harding et al., 2018) mediated through brain systems involved in energy regulation, such as the hypothalamus and metabolic hormones. Generally, endocrine signals, such as ghrelin (Malik et al., 2008; Sun et al., 2015; Rihm et al., 2019) or insulin (Tiedemann et al., 2017), are potential modulators of the reinforcing value of food and its underlying neural processes. Our explorative analyses did not show an effect of nutritive attributes on consumption decisions, but this might change when including individuals' subjective estimation of macronutrients (DiFeliceantonio et al., 2018). In addition, individual dietary restrictions mediated via prefrontal top-down regulation (Hare et al., 2009, 2011) as well as eating habits processed by the dorsal striatum (Lipton et al., 2019) are powerful modulators of food choice and vmPFC signals. It should be noted that food processing in the amygdala may depend on the physiological state (LaBar et al., 2001; Siep et al., 2009). In the present study, all participants met the recommended duration of 12 h of fasting (Smeets et al., 2019). Hunger levels were thus relatively stable across both study days, and individual ratings were not related to our findings. To further elucidate an impact of metabolic state on amygdala activation in the context of ingestive behavior, however, future studies need to directly compare hungry versus sated states.

Previous research has mainly focused on the discrimination between brain networks processing the hedonic properties of food versus those more involved with motivating the desire for food (Castro and Berridge, 2014; Berridge and Robinson, 2016; Pool et al., 2016). How these two mechanisms act in concert to modulate eating behavior, especially in the human brain, has been less addressed (Volkow et al., 2011; Berridge and Kringelbach, 2015). The findings of the present study emphasize that distributed signals in the amygdala to play an important role in both processes. Our multivariate approach may help to identify the impact of hedonic valuation on food decisions and thus explain a relevant amount of variability within a complex neurobehavioral framework of eating behavior. This might be particularly interesting when studying drivers of food decisions in the context of pathologic eating behavior as well as under different metabolic states.
